# Contamination and Health Risk Assessment of Multiple Mycotoxins in Edible and Medicinal Plants

**DOI:** 10.3390/toxins15030209

**Published:** 2023-03-10

**Authors:** Yingyue Zhang, Fengyan Kuang, Chunyao Liu, Kai Ma, Tianyu Liu, Meijuan Zhao, Guangping Lv, He Huang

**Affiliations:** 1 School of Life Science, Nanjing Normal University, Nanjing 210023, China; 2 School of Food Science and Pharmaceutical Engineering, Nanjing Normal University, Nanjing 210023, China; 3 School of Pharmaceutical Sciences, Nanjing Tech University, Nanjing 211816, China; 4 Food Laboratory of Zhongyuan, Nanjing Normal University, Nanjing 210023, China

**Keywords:** mycotoxins, coix seed, lilii bulbus, malt, lotus seed, risk assessment

## Abstract

Edible and medicinal plants (EMPs) are widely used but are easily infected by harmful fungi which produce mycotoxins. Herein, 127 samples from 11 provinces were collected to investigate 15 mycotoxins based on geographic, demographic, processing, and risk characteristics. A total of 13 mycotoxins were detected, and aflatoxin B_1_ (0.56~97.00 μg/kg), deoxynivalenol (9.41~1570.35 μg/kg), fumonisin B_1_ (8.25~1875.77 μg/kg), fumonisin B_2_ (2.74~543.01 μg/kg), ochratoxin A (0.62~19.30 μg/kg), and zearalenone (1.64~2376.58 μg/kg) occurred more frequently. Mycotoxin levels and species were significantly different by region, types of EMPs, and method of processing. The margin of exposure (MOE) values was well below the safe MOE (10,000). AFB_1_ exposure from Coix seed and malt consumption in China was of high health concern. The hazard Index (HI) method showed the range of 113.15~130.73% for malt, indicating a public health concern. In conclusion, EMPs should be concerned because of the cumulative effects of co-occurred mycotoxins, and safety management strategies should be developed in follow-up studies.

## 1. Introduction

The concept of “Let food be thy medicine and medicine be thy food” was widely accepted for thousands of years [1]. In China, many traditional herbs are broadly used as food, and they are usually called medicine and food dual-purpose plants or edible and medicinal plants (EMPs). Currently, there are more than one hundred EMPs listed by the National Health Commission of China, most of which have shown excellent potential for immune or metabolic regulation in numerous studies over the past twenty years [2]. The commonly and frequently used EMPs are *Coicis Semen* (coix seed), *Nelumbinis Semen* (lotus seed), *Hordei Fructus Germinatus* (malt), lilii bulbus, and so on. In some other countries, EMPs are commonly used as dietary supplements or functional foods. Almost 80% of the world population would use them for some part of primary health care [3], and their global market was reported by Allied Market Research (AMR) at about 177.77 billion USD in 2019 and was expected to reach 267.92 billion USD by 2027. Notably, because EMPs do not contain endogenous toxic components, they become more popular for their high safety. However, their exogenous contaminants can be caused by environmental microorganisms, such as *Aspergillus* spp., *Fusarium* spp., and *Penicillium* spp., which can produce mycotoxins [4,5].

Mycotoxins are secondary metabolites of fungi that can be formed on plants in the field or during harvesting, processing, transportation, and storage, and more than 400 species have been identified [6,7]. Aflatoxins (AFs), ochratoxin A (OTA), fumonisins (FBs), deoxynivalenol (DON), T-2 toxin (T-2), HT-2 toxin (HT-2), zearalenone (ZEN), and its derivatives including zearalanone (ZAN), α-zearalenol (α-ZEL), β-zearalenol (β-ZEL), α-zearalanol (α-ZAL), and β-zearalanol (β-ZAL) occur frequently in nature [8,9,10]. Based on their multiple toxicities, many countries have established limits for mycotoxins in various foods intended for human and animal consumption. Recently, concerns about the risk of mycotoxin exposure are increasing as global climate change issues become more acute. Battilani et al. predicted that the risk of AFB_1_ contamination in maize in Europe will increase if global temperatures rise by 2 °C and above [11].

The level of mycotoxins in EMPs may vary depending on the growing, processing, transportation, and storage environments. Since the climatic environment is closely related to geographical locations, there may be geographical differences in the mycotoxin contents of EMPs. Moreover, the substrates that can be used by microorganisms in different EMPs are different, which probably causes differences in mycotoxin levels. A previous study investigated eight mycotoxins of one EMP, Chinese yam, and found that two samples had exceeded the MRL of AFB_1_ [12]. Another study reported patulin contamination and risk assessment in fruit EMPs and found that the average patulin content of dried longan and dried hawthorn was 68.4 mg/kg and 5.1 mg/kg, respectively. Dietary risk assessments suggested that patulin contamination in fruit EMPs posed no public health risk [13], but the cumulative effects of multiple mycotoxins were not investigated. In addition, ginkgo, nutmeg, licorice, fennel, and other EMPs had been investigated for mycotoxin contamination, and the results showed that the contamination was serious [14] but few risk assessments were performed. In addition, some EMPs are traditionally eaten after processing by various means such as steaming with rice wine and roasting, etc., and their effects on mycotoxins are unclear.

On the other hand, because EMPs are rich in carbohydrates, lipids, proteins, and other components such as alkaloids, flavonoids, pigments, etc., the detection of mycotoxins is challenging. A few analytical methods have been established for mycotoxins in EMPs, but they generally cover only one EMP type or are used to analyze a few mycotoxins [15]. Consequently, an analytical method for multiple mycotoxins in a wide range of EMPs is required.

Therefore, a UHPLC-MS/MS method was firstly developed to simultaneously detect fifteen mycotoxins in four kinds of EMPs. After collecting EMP samples (*n* = 127) from their main producing areas in China, the contents of 15 mycotoxins were determined. In addition, mycotoxin contamination levels were investigated according to the species, geographic, and processing characteristics of EMPs. Based on mycotoxin contamination results, exposure, and risk were assessed using the margin of exposure (MOE) and hazard index (HI) methods. The comprehensive study presented here will provide a framework to help identify the contamination signatures of mycotoxins in different EMPs. Furthermore, these results may serve as a scientific basis for risk managers for safety management to minimize exposure to mycotoxins through the consumption of EMPs.

## 2. Results and Discussion

### 2.1. Analytical Method Optimization

**Figure 1:**
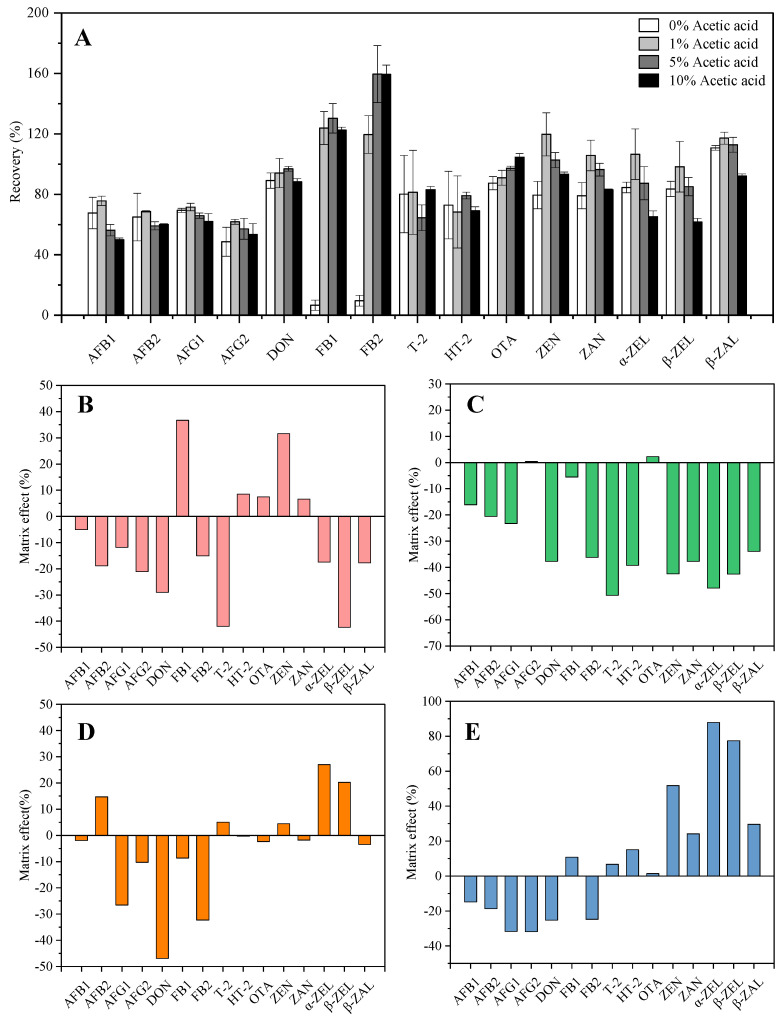
The optimization of extraction solvents of 15 mycotoxins (**A**), and matrix effect investigation of coix seed (**B**), malt (**C**), lotus seed (**D**), and lilii bulbus (**E**) on 15 mycotoxins.

Based on previous studies [16], the solvent for simultaneously extracting multi-mycotoxins was mainly an acetonitrile–water system or an acetonitrile–water plus acid system. Thus, the extraction efficiency of four extraction solvents for 15 mycotoxins was investigated, including 80% acetonitrile containing 0%, 1%, 5%, and 10% acetic acid. From the results of the recovery experiments (Figure 1A), the change in acid concentration had a great influence on the extraction rate of mycotoxins, especially FB_1_ and FB_2_. After the addition of acetic acid, the recoveries of FB_1_ and FB_2_ increased from 6.67% and 9.63% to 123.89% and 119.58%, respectively. This may be because FB_1_ and FB_2_ have lots of carboxyl groups, which have requirements on the acidity of the organic extraction solvent. Recoveries of ZEN, ZAN, α-ZEL, β-ZEL, and β-ZAL were the best when containing 1% acetic acid which began to decrease after the acid concentration was further increased. Most mycotoxins showed acceptable recoveries (61.88~123.89%) when using 80% acetonitrile containing 1% acetic acid, and thus, it was selected as the best extraction solvent.

### 2.2. Analytical Method Validation

#### 2.2.1. Matrix Effect and Linearity

UHPLC-MS/MS chromatograms of the mixed standards of 15 mycotoxins are shown in Figure S1, and MS quantification parameters are shown in Table S1. In general, ME% within ±20% was classified as not influenced by matrix, whereas ME values < −20% or > +20% indicate strong matrix effects [17]. As shown in Figure 1, the four EMPs exhibited different matrix effects on 15 mycotoxins. The matrix effects of coix seed (Figure 1B), lotus seed (Figure 1D), and lilii bulbus (Figure 1E) on 15 mycotoxins showed some enhancement and some inhibition, whereas malt (Figure 1C) was mostly inhibiting. Although the extraction solution was purified by QuEChERS, some substances that interfered with the target analyte remained in the solution, which was consistent with previous studies [18]. Due to the strong matrix effect on some mycotoxins, reliable results and accurate quantification in the four EMP samples were carried out using matrix-matched calibration curves to compensate for ME. The linearity of mycotoxins in different EMPs is shown in Table 1. The linear relationships of 15 mycotoxins in matrix-matched calibration curves of coix seed, malt, lotus seed, and lilii bulbus were good (*R*^2^ > 0.99).

#### 2.2.2. Accuracy, Precision, LOD, and LOQ

Since many mycotoxin contents of EMPs were not clear, the Chinese Food Industry Standard (LS/T 6133-2018) was regarded as a reference for a spiking experiment. As shown in Table 2, the recoveries for 15 mycotoxins were 62.0~113.5% in coix seed, 65.5~118.8% in malts, 61.5~116.3% in lotus seed, and 61.0~118.8% in lilii bulbus, which were within the acceptable ranges for mycotoxins analysis. In addition, the precisions for 15 mycotoxins were 1.6~19.6% in coix seed, 0.8~15.8% in malts, 1.0~18.5% in lotus seed, and 0.7~18.3% in lilii bulbus. The above results showed acceptable accuracy and precision of this method. The ranges of LODs and LOQs were 0.09~15 μg/kg and 0.25~50 μg/kg, respectively. They were well below the MRL set by the Chinese regulatory limit of the mycotoxin (national food safety standard/standards for limits of mycotoxins in food/GB2761-2017) and Commission Regulation (EC) no. 401/2006 [19] and were comparable to or lower than those of previous studies for mycotoxin analysis [18].

### 2.3. Detection of Mycotoxins in the Edible and Medicinal Plants

#### 2.3.1. Occurrence of Mycotoxins in Coix Seed

Among the four EMPs, coix seed was the most serious one contaminated by mycotoxins. As shown in Table 3, 12 mycotoxins were detected in the coix seed samples (*n* = 50), and their order of occurrence was ZEN (100%) > FB_1_ (60%) > FB_2_ (40%) > AFB_1_ (28%) > DON (18%) > OTA = ZAN (12%) > α-ZEL = β-ZEL (6%) > AFB_2_ = T-2 = HT-2 (2%). According to the Chinese regulatory limit of the mycotoxin (national food safety standard/standards for limits of mycotoxins in food/GB2761-2017), the MRL was 5 μg/kg for AFB_1_, 1000 μg/kg for DON, 5 μg/kg for OTA, and 60 μg/kg for ZEN. The AFB_1_ level in the coix seeds ranged from 0.14 to 19.54 μg/kg with a mean concentration of 4.55 μg/kg, and three samples (6%) exceeded the MRL. The DON level in the coix seeds ranged from 22.97 to 1570.35 μg/kg with a mean concentration of 412.36 μg/kg, and two samples (4%) exceeded the MRL, which was higher than previous studies [20]. As for OTA, there were no samples exceeding the MRL, and the content range was 1.37~2.10 μg/kg with a mean concentration of 1.75 μg/kg. However, worryingly, all coix seed samples (100%) were contaminated with ZEN, and the range of the ZEN was 1.64~2376.58 μg/kg with a mean concentration of 131.85 μg/kg, which was higher than the MRL (60 μg/kg). Furthermore, there were 14 samples (28%) that exceeded the MRL. Likewise, the high occurrences of ZEN in the coix seeds were also previously reported as 70% [21], 84.62% [22], and 98.7% [20]. The maximum levels of FB_1_ and FB_2_ were 1875.77 μg/kg and 543.01 μg/kg, respectively. In this study, the contamination of FB_1_ and FB_2_ in the coix seeds was severe, which was consistent with the previous study [20].

As shown in Table S2, samples collected from Guizhou were the most contaminated with an 18% over-MRL rate, whereas Heilongjiang was the least polluted with a 6% over-MRL rate. The annual average temperature in Guizhou and Heilongjiang was 16.4 °C and 4.0 °C, and the average humidity was 80% and 70%, respectively. Previous studies had identified the fungal community composition of coix seeds collected from Guizhou [23] and found that the dominant fungi were *Aspergillus*, *Penicillium*, and *Fusarium*, which can produce mycotoxins. Taking *Fusarium* as an example, it produces ZEN at 15~17 °C and DON at 15~30 °C [24], and this condition was exactly the temperature in Guizhou. In addition, the storage period of the coix seeds is in the rainy season in Guizhou, which could accelerate contamination. However, the planting area of the coix seeds in Guizhou ranks first in China, and the output accounts for two-thirds of the total, indicating mycotoxin regulation of coix seeds on the market needs to be carried out.

#### 2.3.2. Occurrence of Mycotoxins in Malt

A total of eight mycotoxins were detected in malt samples (n = 46), and their order of occurrence was DON (52.2%) > OTA (15.22%) > ZEN (8.70%) > AFB_1_ = T-2 (6.52%) > AFB_2_ = FB_1_ = HT-2 (2.17%). As shown in Table 3, the DON level in malts ranged from 15.49 to 1100.88 μg/kg with a mean concentration of 94.06 μg/kg, and one sample exceeded the MRL (1000 μg/kg). Malt is the product of germination from barley and is also a raw material used in the beer industry. A previous study reported that DON was the most frequently polluted mycotoxin in barley with a rate of 94%, and the DON concentration ranged from 1700 to 7500 μg/kg in barley samples [25]. However, in the present study, the content of DON in most malt samples was far below this level. Another study showed that the DON content decreased by up to 80% during malting, and this may be due to the good water solubility of DON, which was transferred to the water during the soaking process of barley germination [26]. For AFB_1_ analysis, the range in the malt was 5.14~97.00 μg/kg, and all AFB_1_-positive samples exceeded the MRL (5 μg/kg), with an average concentration of 36.13 μg/kg. Due to the high toxicity of AFB_1_, it has been classified as a carcinogenic compound (group 1) by the International Agency for Research on Cancer (IARC); therefore, AFB_1_ contamination in malt calls for vigilance. The ZEN content in malt ranged from 2.86 to 92.02 μg/kg with a mean level of 46.64 μg/kg, and two samples (4.35%) exceeded the MRL (60 μg/kg). Moreover, the detection rate of OTA was relatively high (15.22%), but there were no samples that exceeded the MRL (5 μg/kg). The concentration range of T-2 in the malt was 3.09~56.08 μg/kg, with a mean level of 21.24 μg/kg. For the remaining three mycotoxins, only one positive sample was detected for each mycotoxin at levels of 2.04 μg/kg for AFB_2_, 49.65 μg/kg for FB_1_, and 260 μg/kg for HT-2.

#### 2.3.3. Occurrence of Mycotoxins in Lotus Seed

There were only two mycotoxins, AFG_2_ and OTA, detected in the lotus seed samples (*n* = 15), and their occurrences were both 13.33% (Table 3). The content of AFG_2_ in the lotus seeds was from 1.15 to 1.45 μg/kg, and the average level was 1.30 μg/kg. The OTA content in the lotus seeds ranged from 1.95 to 2.03 μg/kg, with a mean level of 1.99 μg/kg. Multiple mycotoxins were not detected simultaneously in the lotus seed samples. In this study, the mycotoxin contamination of lotus seeds was the lightest among the four EMPs. This may be due to the fact that most of the commercial lotus seeds we collected had been polished, whereas mycotoxins are mostly found in the epidermis of the seeds [27].

#### 2.3.4. Occurrence of Mycotoxins in Lilii Bulbus

FB_1_, HT-2, and OTA were detected in lilii bulbus (*n* = 16), and their incidence rates were 81.25%, 37.50%, and 25%, respectively (Table 3). The range of concentration was 10.49~22.06 μg/kg for FB_1_, 73.15~267.50 μg/kg for HT-2, and 1.27~19.30 μg/kg for OTA. Additionally, the mean concentrations of FB_1_, HT-2, and OTA were 15.29 μg/kg, 174.21 μg/kg, and 7.11 μg/kg, respectively. There was one sample whose OTA content exceeded the MRL (5 μg/kg). To our knowledge, this is the first reveal of mycotoxin contamination in lilii bulbus. Previous research on lilii bulbus focused on its volatile constituents, polysaccharides, and anti-inflammatory activity. In recent years, a few studies have focused on its safety and carried out exposure assessments of heavy metal in lilii bulbus and found that Cd pollution was serious [28,29]. Our study indicated that FB_1_ occurred at a high frequency but at a low level in lilii bulbus, whereas HT-2 was the opposite with a high content of 267.50 μg/kg. In addition, there were cases where the OTA content exceeded the MRL.

### 2.4. Effects of Processing Treatment on Mycotoxins in the Edible and Medicinal Plants

#### 2.4.1. Comparison between Coix Seed and Roasted Coix Seed

**Figure 2:**
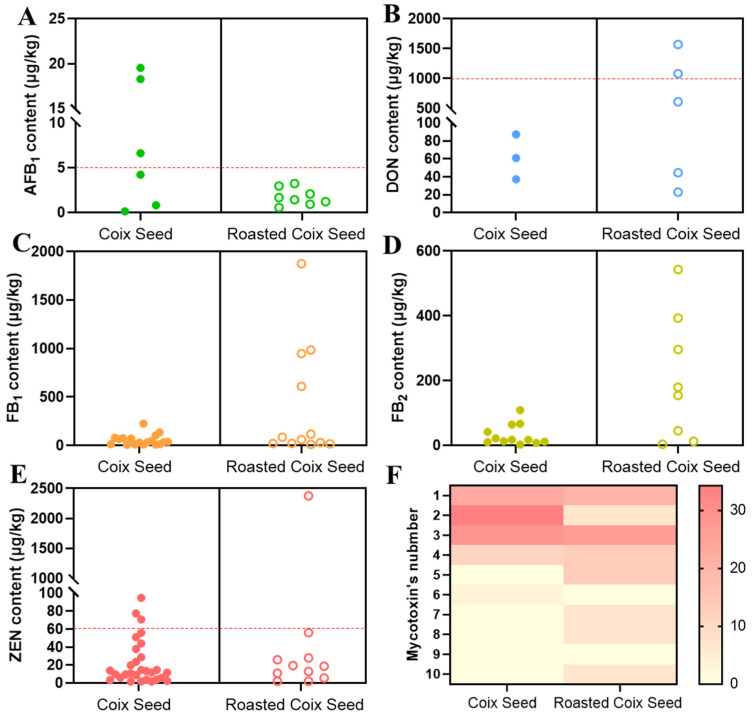
Comparison of the concentrations of AFB_1_ (**A**), DON (**B**), FB_1_ (**C**), FB_2_ (**D**), and ZEN (**E**), and mycotoxin co-occurrence in unroasted coix seeds and roasted coix seeds (**F**). The red dotted line represents the Chinese regulatory limit (national food safety standard/standards for limits of mycotoxins in food/GB2761-2017) of the mycotoxin. In the F graph, the redder the color, the higher the frequency of occurrence.

Another traditional way of using coix seeds in China is to roast them, which may cause changes in mycotoxins. Therefore, we further compared the content and types of mycotoxins in unroasted coix seeds and roasted coix seeds. As shown in Figure 2, AFB_1_, DON, FB_1_, FB_2_, and ZEN were detected in both unroasted coix seeds and roasted coix seeds. The content of AFB_1_ in unroasted coix seeds exceeded the MRL (5 μg/kg), but not in roasted coix seeds (Figure 2A). Previous studies have shown that AFB_1_ levels decreased by 40~80% after roasting due to the hydrolysis of the lactone ring [30], and this may be the reason for the lower content in roasted coix seeds. However, the detection of DON showed the opposite result (Figure 2B). Similarly, the content of FB_1_ in roasted coix seeds was significantly higher than that in unroasted coix seeds (Figure 2C), and samples containing high concentrations of FB_2_ were detected in roasted coix seeds (Figure 2D). In addition, samples exceeding the MRL (60 μg/kg) of ZEN were detected in both unroasted coix seeds and roasted coix seeds, and the sample heavily contaminated by ZEN with a content of 2376.58 μg/kg was roasted coix seeds (Figure 2E). Interestingly, all the roasted coix seeds with mycotoxins detected were from Guizhou, but their content distributions of DON, FB_1_, FB_2_, and ZEN showed higher dispersion than those without roasting, indicating that there were still other factors affecting mycotoxin levels besides geography and processing. The reason for this phenomenon may be that these roasted coix seeds had undergone different storage times or storage conditions before being processed. The humid climate of Guizhou may be one of the reasons to promote the production of mycotoxins during storage. On the other hand, this result may also be caused by the manufacturers. Because of the bad appearance and smell of severely moldy coix seeds, manufacturers improved the situation by roasting them and continued to sell them for profit. Interestingly, α-ZEL and β-ZEL, the two derivatives of ZEN, were detected only in three roasted coix seed samples (6%) but not in the unroasted coix seeds. Very coincidentally, these three samples also contained high levels of FB_1_, FB_2_, DON, ZEN, and ZAN, implying that mildew was terrible. This verified that the manufacturer may sell the moldy coix seed by roasting. Generally, α-ZEL and β-ZEL are formed during phase II metabolism in plants (glucosides) or animals and humans (glucuronides). During coix seed roasting, carbohydrates undergo Maillard reactions, which may have contributed to α-ZEL and β-ZEL exposure. Notably, studies have shown that α-ZEL exhibits a higher estrogenic effect than ZEN [31], suggesting that roasted coix seed may increase this risk. Besides, as shown in Figure 2F, results showed that the co-occurrence of mycotoxins in roasted coix seed was more serious than in unroasted coix seeds, and at most, 10 kinds of mycotoxins could be simultaneously detected in one roasted coix seed sample.

#### 2.4.2. Comparison of Malt, Roasted Malt, and Dark-Roasted Malt

**Figure 3:**
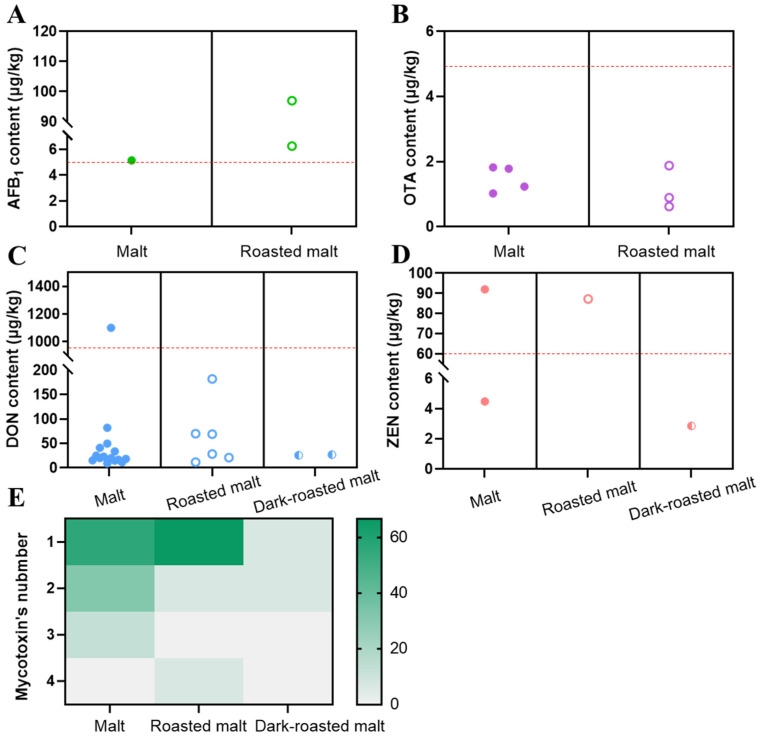
Comparison of the concentrations of AFB_1_ (**A**), DON (**B**), OTA (**C**), and ZEN (**D**), and mycotoxin co-occurrence in malt, roasted malt, and dark-roasted malt (**E**). The red dotted line represents the Chinese regulatory limit (national food safety standard/standards for limits of mycotoxins in food/GB2761-2017) of the mycotoxin. In the (**E**) graph, the greener the color, the higher the frequency of occurrence.

Traditionally, malt slightly roasted to yellow could become roasted malt, and when continuously roasted until browned, it could become dark-roasted malt, and they are all widely used in China. As shown in Figure 3, AFB_1_ and OTA were detected in both malt and roasted malt, and DON and ZEN occurred in all three types of malt. The concentration of AFB_1_ in all positive samples (6.52%) exceeded the MRL (5 μg/kg), and it was higher in roasted malt samples at 97.00 μg/kg (Figure 3A). The OTA content of the unroasted malt and roasted malt was similar, and neither exceeded the MRL (Figure 3B). In Figure 3C, the DON content of one malt sample exceeded the MRL, but from the average level, the order of samples according to the DON concentration was roasted malt (103.12 μg/kg) > unroasted malt (98.86 μg/kg) > dark-roasted malt (26.42 μg/kg). The order of the samples was unroasted malt > roasted malt > dark-roasted malt based on ZEN concentration (Figure 3D). Furthermore, the co-occurrence frequency of mycotoxins in dark-roasted malt was the lowest (Figure 3E). Only one mycotoxin was detected in both unroasted malt and roasted malt, but up to four mycotoxins could be detected simultaneously in roasted malt. The higher temperature and longer time that were required to produce dark-roasted malt may decrease mycotoxins, and it was proved that mycotoxin destruction was dependent on both the temperature and the duration of exposure. For example, the degradation of AFB_1_ and AFB_2_ was positively correlated with temperature and time during pistachio nut roasting [32]. On the other hand, prolonged high-temperature treatment resulted in changes in the chemical composition of malt and a dramatic decrease in water activity (*a*_w_), creating an environment in which microorganisms are difficult to grow. Therefore, dark-roasted malt is also less likely to be contaminated with mycotoxins during storage.

### 2.5. Estimation of the Mycotoxin Exposure

EMPs are highly consumed in China because they have both the nutritional quality of food and the function of medicine. Mycotoxin contamination in EMPs has been reported recently but few risk assessments have been performed. Therefore, we calculated exposure levels for males and females in China based on the average contents of mycotoxins in positive samples that were detected in this study. The Joint FAO/WHO Expert Committee on Food Additives (JECFA) established the Provisional Maximum Tolerable Daily Intake (PMTDI) for DON, FBs (single or sum), T-2/HT-2 (single or sum), ZEN and its derivatives (single or sum), and OTA [33]. The PMTDI values for these mycotoxins are listed in Table 4. Due to the high toxicity of AFs, their PMTDI was not established. From the overall result, females have higher levels of mycotoxin exposure than males, owing to the exposure per kg bodyweight being higher for females [34]. Among the four EMPs, the consumption of coix seeds produced the highest exposure to mycotoxins including DON, FB_1_, FB_2_, T-2, ZEN, ZAN, α-ZEL, and β-ZEL, but none of their exposure levels exceeded the PMTDIs. Additionally, the exposure of ZEN derivatives involving ZAN, α-ZEL, and β-ZEL was only found in coix seeds. As for malt, the exposure of AFB_1_ and HT-2 was the highest in the four EMPs. The exposures of AFB_1_ and HT-2 in malt were 8.19 and 58.91 ng kg^−1^ b.w. day^−1^ for males, and 9.46 and 68.06 for females. It was worth noting that exposure to HT-2 for females had exceeded the PMTDI (60 ng kg^−1^ b.w. day^−1^), indicating a potential health risk. Lilii Bulbus showed a higher exposure of OTA than the other three EMPs, although it did not exceed the PMTDI (14 ng kg^−1^ b.w. day^−1^), and mycotoxins exposure levels from lotus seeds were generally low.

### 2.6. Risk Assessment

#### 2.6.1. Risk Characterization Using the MOE Approach

**Figure 4:**
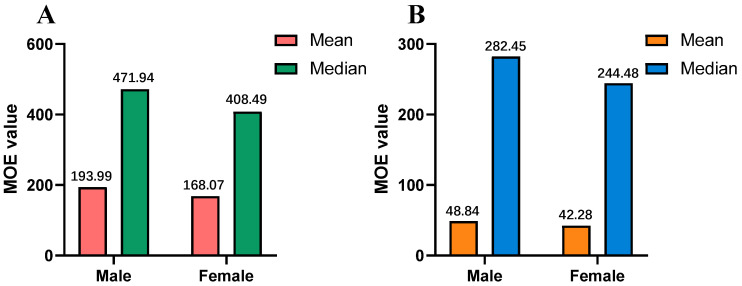
Margin of exposure (MOE) values from coix seed (**A**) and malt (**B**) consumption for males and females.

We conducted MOE risk assessments from the mean and median levels of AFB_1_ contamination to assess risk exposure. As shown in Figure 4, all MOE values for coix seed and malt consumption were below the safety margin of 10,000 for both male and female populations. The MOE values obtained from males and females due to the consumption of coix seeds were 193.99 and 168.07 at the mean and 471.94 and 408.49 at the median, respectively (Figure 4A). Moreover, The MOE values obtained from males and females due to the consumption of coix seeds were 48.84 and 42.28 at the mean and 282.45 and 244.48 at the median, respectively (Figure 4B). Obviously, the probabilistic results for MOE were lower than 10,000 for all population groups, which implies that the consumption of coix seeds and malt contaminated with AFB_1_ entails a risk to public health. The consumption of coix seeds and malt was referenced in the data in the Chinese Pharmacopoeia. However, in real life, it may be much higher than this. Because these two EMPs have health functions, people usually take them for a long time for therapeutic purposes. The MOE values obtained in this study were similar to or lower than those reported in other studies. The EFSA conducted a risk assessment of aflatoxins in food in 2020, and the MOE values for AFB_1_ exposure ranged from 5000 to 29 [35]. Zhang et al. assessed the probabilistic risk of dietary exposure to AFB_1_ contamination in foodstuffs in Guangzhou, China, using the MOE method, and found that the range of MOE was 4020~50 [36]. Another study from Pakistan reported that MOE values for AFB_1_ exposure in rice and wheat were 112.9~13.2 [37]. AFB_1_ is a highly toxic and strongly carcinogenic contaminant, and it causes serious liver damage and is very likely to cause hepatocellular carcinomas (HCCs). The AFB_1_ risk from coix seed and malt consumption in China should be considered a priority for risk management.

#### 2.6.2. Risk Characterization Using the HI Approach

**Figure 5:**
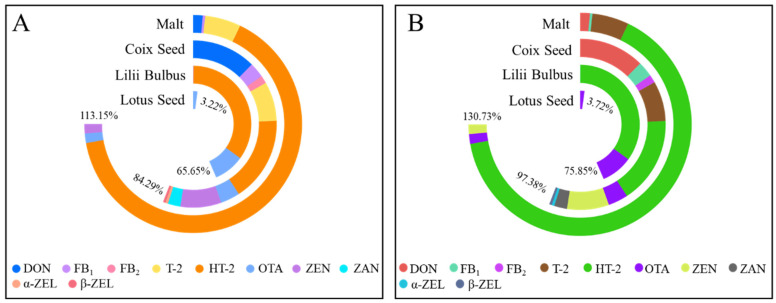
Hazard quotients (HQ) and hazard index (HI) of detected mycotoxins in coix seed, malt, lotus seed, and lilii bulbus for males (**A**) and females (**B**). The length of different colors on each ring represents the value of HQ, and HI is the sum of the HQs.

Based on the results of mycotoxin exposure, a cumulative risk assessment was carried out by the hazard quotient (HQ) and hazard index (HI) methods. HI is the sum of HQs, and when the HI exceeds 100%, it indicates that there might be a high risk in the concerned food to the target population. Figure 5A,B represents the cumulative risks in the four EMPs for males and females, respectively. The cumulative risk of multiple mycotoxins in lotus seeds was the lowest, with a value of 3.22% for males and 3.72% for females. Thence, there was no health risk to mycotoxins when consuming lotus seeds because the calculated HIs were well below 100%. Moreover, the HIs of lilii bulbus were 65.65% and 75.85% for males and females, respectively, and HT-2 contributed the most to the HI. For coix seeds, the composition of its HI was the most complex among the four EMPs. The HIs for males and females were 84.29% and 97.38%, respectively. The order of the contribution of mycotoxins to the total risk was HT-2 > DON > ZEN > T-2 > OTA > FB_1_ > ZAN > FB_2_ > α-ZEL > β-ZEL. In the investigation of the mycotoxin concentration in coix seeds, ZEN was the one with the highest occurrence and the highest rate of exceeding the MRL, but not the one with the highest contribution rate to HI in the risk assessment. As the setting of PMTDI will take into account the toxicology data of mycotoxins, there are differences between the PMTDI values of different mycotoxins. Therefore, the HQ calculated from the PMTDI value would be different even if the exposure of the two mycotoxins was the same. Additionally, in the risk assessment of malt, the HIs of multiple mycotoxins were 113.15% for males and 130.73% for females which all exceeded 100%, implying that there was a risk of mycotoxin exposure when intaking malt. The order of the contribution of mycotoxins to HI was HT-2 > T-2 > OTA > DON > ZEN > FB_1_. This was consistent with the risk assessment results of coix seeds, with HT-2 showing the highest HQ.

The EMPs are different from ordinary herbal medicines in that they have the properties of food, so they are used in a wider range and are consumed more frequently. Therefore, in reality, the risk of multiple mycotoxin exposure from EMPs may be higher than calculated in our study. Lu et al. took 500 g/day as a daily intake, and the HI of mycotoxins in the coix seeds was 958.72%, which was much higher than the 84.29~97.38% calculated in this study [22]. Other studies also highlighted the need to monitor mycotoxins in coix seeds [20].

## 3. Conclusions

This study highlights the importance of assessing the cumulative health risks caused by multiple mycotoxins on EMPs. A high risk of mycotoxin exposure from coix seeds and malt was warned, with particular emphasis on the urgency for the risk management of AFB_1_. Moreover, AFG_1_ and β-ZAL were not detected in all EMP samples, indicating a lesser risk. Of special note was the fact that processing results in the appearance of mycotoxin derivatives, suggesting that it was also necessary to emphasize the control of the processing procedure. The mycotoxin with the highest frequency in different EPMs was different, and it may require more explicit limits for specific EMPs. The use and demand of EMPs have increased sharply, and the data on the risk of mycotoxin exposure are staggering, so we think this is a field of research that should be paid more attention to in the future.

## 4. Materials and Methods

### 4.1. Sample Collection

From 2020 to 2022, a total of 127 samples from their main production areas were collected, including 50 of coix seeds, 46 of malt, 15 of lotus seeds, and 16 of lilii bulbus, and these four kinds of the EMP were frequently used in China. In addition, there were 15 roasted coix seeds among the 50 coix seed samples. Additionally, there were 15 roasted malts and 15 dark-roasted malts among the 46 malt samples. All samples were stored at −20 °C and ground into powder by a milling machine (JC-FW-100, Shandong, China) with a sieve of trapezoid holes of 1.00 mm before analysis.

The main production areas and their collection rates of coix seeds were Heilongjiang (9/50), Guizhou (32/50), and Yunnan (9/50) provinces; malt were Hebei (25/46), Sichuan (5/46), and Anhui (16/46) provinces; lotus seeds were Hunan (5/15), Fujian (5/15), and Jiangxi (5/15) provinces; and lilii bulbus were Gansu (5/16), Jiangsu (6/16), and Hunan (5/16) provinces. To investigate the contamination characteristics of mycotoxins, we divided these provinces into northern and southern areas based on geographic factors. Heilongjiang, Hebei, and Gansu provinces belonged to the northern area, and Guizhou, Yunnan, Sichuan, Anhui, Hunan, Fujian, and Jiangxi provinces belonged to the southern area. Additionally, geographical information on these areas, including average temperatures, relative humidity (RH), and latitudes are shown in Table S2.

### 4.2. Chemicals and Reagents

Reference substances of aflatoxin B_1_ (AFB_1_), aflatoxin B_2_ (AFB_2_), aflatoxin G_1_ (AFG_1_), aflatoxin G_2_ (AFG_2_), deoxynivalenol (DON), fumonisin B_1_ (FB_1_), fumonisin B_2_ (FB_2_), T-2 toxin (T-2), HT-2 toxin (HT-2), ochratoxin A (OTA), zearalenone (ZEN), zearalanone (ZAN), α-zearalenol (α-ZEL), β-zearalenol (β-ZEL), and β-zearalanol (β-ZAL) were purchased from Sigma Aldrich Chemicals (St. Louis, MO). Standard stock solutions (100 µg/mL) were diluted to mixed working solutions with 35% methanol and sample matrix solutions. Concentrations of the mixed standard working solutions are shown in Table S3.

Methanol (LC/MS grade) and acetonitrile (LC grade) were purchased from Merck KGaA (Darmstadt, Germany). Formic acid, acetic acid, and ammonium acetate, both of LC/MS grade, were supplied by Aladdin Biochemical Technology Co., Ltd. (Shanghai, China). QuEChERS purifier was obtained from Meizheng Bio-Tech Co., Ltd. (Wuxi, Jiangsu, China), and it consisted of octadecyl silica (C18), silica, graphitized carbon black (GCB), magnesium sulfate (MgSO_4_), and primary secondary amine (PSA).

### 4.3. Sample Preparation

An amount of 1.0 g sample was weighed, and 20 mL of 80% acetonitrile solution (containing 1% acetic acid) was added. After standing for 1 h, the mixture was shaken for 2 min on an MX-S Vortex (DLAB Scientific, Beijing, China), and it was subsequently centrifuged at 8000 rpm for 5 min. Next, 10 mL of supernatant was transferred to another PTFE centrifuge tube, and the QuEChERS purifier including 240 mg of MgSO_4_, 80 mg of PSA, 80 mg of C18, 80 mg of silica, and 25 mg of GCB was added, and it was shaken for 2 min. Successively, it was centrifuged at 8000 rpm for 5 min, and 5 mL of supernatant was dried with nitrogen at 50 °C. Finally, the residue was re-dissolved in 1.0 mL of 35% methanol solution and filtered through a 0.22 μm nylon syringe filter before UHPLC-MS/MS analysis.

### 4.4. UHPLC-MS/MS Analysis

The UHPLC-MS/MS analysis was performed on a Shimadzu LCMS 8050 system and the analytes were separated with a Shim-pack GIST RP-C18 column (2.1 mm × 100 mm i.d., 2 μm) (Shimadzu, Kyoto, Japan). The column was eluted with mobile phase A (0.1% formic acid and 1 mM ammonium acetate) and B (methanol) at 0.2 mL/min with 1.0 µL injections, and the oven temperature was 40 °C. The gradient started with 20% B, increased linearly from 20% to 95% B in 5 min, was maintained at 95% B for 4 min, and then decreased linearly from 95% to 20% in 2 min. In total, 15 mycotoxins were identified by multiple reaction monitoring (MRM) in both the electrospray ionization (ESI) positive and negative modes. The capillary voltage (4.0 kV), source temperature (300 °C), desolvation temperature (526 °C), the flow rate of nebulizing gas (3.0 L/min), and heating gas (10 L/min) had been all optimized. The MRM transitions of 15 mycotoxins and MS parameters were presented in Table S1.

### 4.5. Analytical Method Validation

To validate the QuEChERS-UHPLC-MS/MS method, we evaluated the linearity, matrix effect, limit of detection (LOD), limit of quantification (LOQ), accuracy, and precision. Linearity was assessed by the correlation coefficient (*R*^2^) of the matrix-matched calibration curves. Matrix effects (ME) were calculated by the slope ratios of matrix-matched calibration curves and pure solvent for 15 mycotoxins. The signal-to-noise (S/N) ratio of 3:1 was considered as the LOD and 10:1 as the LOQ. Recovery experiments were performed to estimate the accuracy of the method with three spiked levels in three replicates. The relative standard deviations (RSDs) were evaluated from the recovery test of three replicates for individual mycotoxin, and the RSDs represented the precision of the QuEChERS-UHPLC-MS/MS method.

### 4.6. Exposure Analysis

The exposure of mycotoxin was calculated by the mean level of mycotoxin obtained from this study, the EMP consumption data, and the mean body weight. According to the Pharmacopoeia of The People’s Republic of China, the maximum daily intake of coix seeds is 30 g, malt is 15 g, lotus seeds is 15 g, and lilii bulbus is 12 g. Moreover, the average body weight was 66.2 kg for males and 57.3 kg for females on the basis of the Report on Nutrition and Chronic Diseases in Chinese Residents. The calculation formula for exposure is as follows:

(1) $$\mathrm{Exposure}=\frac{\mathrm{Mycotoxin}\;\mathrm{Content}\;\times \;\mathrm{EMPs}\;\mathrm{Consumption}}{\mathrm{Body}\;\mathrm{Weight}}$$

### 4.7. Risk Assessment

As AFB_1_ is a genotoxic carcinogen with no threshold value, and has a different risk at any exposure level, there is no provisional maximum tolerable daily intake (PMTDI) value for it. Herein, we evaluated the risk for AFB_1_ by the margin of exposure (MOE) method, and for other mycotoxins, by the hazard index (HI) method.

#### 4.7.1. Margin of Exposure (MOE) Approach

The MOE method was proposed by the European Food Safety Authority (EFSA) to analyze the risk of compounds that are both genotoxic and carcinogenic [38]. MOE was used to assess the risk of AFB_1_ intake in the population using animal studies or population studies deduced to cause primary hepatocellular carcinoma as the toxic effect endpoint. The formula for calculating the MOE value is shown below.

MOE = BMDL_10_/Exposure (2)

BMDL_10_ was the benchmark dose lower confidence limit of 10% extra risk. Based on animal carcinogenicity data, the EFSA-calculated BMDL_10_ for AFB_1_ was 400 ng kg^−1^ body weight day^−1^ [35]. When MOE > 10,000, the hazardous substance can be considered as a low risk to human health and a low public health concern. When MOE < 10,000, it can be considered as a high public health concern. MOE values cannot quantify the risk but indicate a level of concern, which means that the lower the MOE value, the higher the level of concern [38].

#### 4.7.2. Hazard Index (HI) Approach

For mycotoxins other than AFB_1_, the cumulative risk assessment of multi-mycotoxins was performed by the PMTDI, hazard quotients (HQ), and hazard index (HI) methods, and their calculation formulas were as follows. If the HI exceeds 100%, the cumulative exposure of multi-mycotoxins has exceeded the maximum acceptable level, and thus there might be a risk.

(3) $$\mathrm{Hazard}\;\mathrm{Quotients}\;(\mathrm{HQ})=\frac{\mathrm{Exposure}\;\mathrm{of}\;\mathrm{the}\;\mathrm{Concerned}\;\mathrm{Mycotoxin}}{\mathrm{Provisional}\;\mathrm{Maximum}\;\mathrm{Tolerable}\;\mathrm{Daily}\;\mathrm{Intake}\;(\mathrm{PMTDI})}$$

(4) Hazard Index (HI)=∑HQ

## Figures and Tables

**Table 1:** Linearity of mycotoxins in different edible and medicinal plants.

Mycotoxin	Coix Seed		Malt		Lotus Seed		Lilii Bulbus	
	Linear Equation	*R* ^2^	Linear Equation	*R* ^2^	Linear Equation	*R* ^2^	Linear Equation	*R* ^2^
AFB_1_	y = 89,008x + 3968.1	0.9994	y = 82,676x − 1422.8	0.9999	y = 90,462x − 11,672	0.9991	y = 74,386x + 2183.0	0.9995
AFB_2_	y = 153,518x − 1061.7	0.9987	y = 138,322x − 5133.0	0.9982	y = 147,594x + 6408.4	0.9975	y = 139,854x − 996.0	0.9999
AFG_1_	y = 38,672x − 1117.6	0.9996	y = 32,578x + 929.45	0.9969	y = 30,059x − 673.87	0.9987	y = 31,205x − 3836.4	0.9994
AFG_2_	y = 71,915x + 199.57	0.9998	y = 61,116x + 4006.0	0.9997	y = 64,511x + 12,303	0.9995	y = 45,148x + 1254.1	0.9989
DON	y = 1569.6x + 24,926	0.9995	y = 1379.2x + 14,256	0.9999	y = 1174.5x + 4571.9	0.9998	y = 1652.6x + 19,961	0.9994
FB_1_	y = 2231.7x − 923.02	0.9994	y = 2237.4x + 3494.5	0.9995	y = 1901.0x + 2709.0	0.9995	y = 2474.5x + 10,971	0.9975
FB_2_	y = 2347.6x + 549.58	0.9941	y = 2212.7x + 3645.2	0.9997	y = 2020.5x + 733.65	0.9996	y = 2601.8x − 1422.7	0.9981
T-2	y = 32,404x + 13,794	0.9987	y = 25,705x − 8107.7	0.9974	y = 34,427x − 6006.7	0.9972	y = 30,629x − 7210.0	0.9976
HT-2	y = 6913.6x + 1146.8	0.9983	y = 5962.7x − 6668.1	0.9991	y = 6249.8x + 10,376	0.9987	y = 7455.0x − 4769.7	0.9964
OTA	y = 43,708x + 1632.1	0.9994	y = 41,607x + 4557.4	0.9987	y = 39,728x − 4309.9	0.9984	y = 41,260x + 5233.1	0.9997
ZEN	y = 7836.8x + 13,245	0.9998	y = 7414.3x + 14,432	0.9994	y = 6976.7x + 6915.9	0.9999	y = 9206.9x + 10,960	0.9982
ZAN	y = 12,334x − 9184.7	0.9998	y = 11,747x + 13,984	0.9999	y = 11,012x − 1046.5	0.9997	y = 12,568x − 5312.8	0.9999
α-ZEL	y = 3017.5x − 273.47	0.9989	y = 2979.4x + 290.48	0.9999	y = 3011.2x − 5560.3	0.9996	y = 2692.9x − 2527.1	0.9999
β-ZEL	y = 3140.0x − 1017.1	0.9976	y = 3065.4x + 1546.5	0.9995	y = 3064.5x − 5767.7	0.9997	y = 2621.0x − 60.477	0.9995
β-ZAL	y = 3117.6x + 3628.1	0.9996	y = 3305.9x + 9154.8	0.9993	y = 2825.9x + 4687.3	0.9978	y = 3845.8x − 4362.7	0.9994

**Table 2:** Recovery (*n* = 3), precision (*n* = 3), LOD, and LOQ of mycotoxins in different edible and medicinal plants.

Mycotoxin	Spiked Level (μg/kg)	Coix Seed		Malt		Lotus Seed		Lilii Bulbus		LOD (μg/kg)	LOQ (μg/kg)
		Recovery (%)	Precision (%)	Recovery (%)	Precision (%)	Recovery (%)	Precision (%)	Recovery (%)	Precision (%)		
AFB_1_	2	99.1	19.6	68.1	6.1	81.6	4.0	66.3	8.2	0.3	1
	10	108.1	7.3	67.1	9.6	76.6	4.4	62.1	10.6		
	20	110.0	4.3	82.0	14.3	67.7	10.4	61.2	6.8		
AFB_2_	1	94.6	15.0	88.6	9.0	66.3	1.9	61.6	13.2	0.09	0.25
	5	86.4	9.6	73.1	7.9	63.6	8.7	64.9	5.7		
	10	102.7	3.1	88.1	1.1	65.0	15.8	61.0	8.2		
AFG_1_	2	105.4	13.3	87.3	6.0	84.8	1.0	63.5	11.5	0.3	1
	10	92.5	10.8	80.9	6.7	76.5	1.9	62.7	8.9		
	20	85.2	17.5	101.7	1.9	69.5	1.3	61.6	6.5		
AFG_2_	1	104.7	15.3	66.3	15.0	81.1	18.0	83.6	17.4	0.09	0.25
	5	113.5	7.5	74.9	12.1	85.1	10.6	89.0	0.9		
	10	108.2	6.2	74.8	15.8	116.3	18.5	65.2	5.5		
DON	150	62.4	5.6	65.5	11.7	97.0	5.3	77.4	2.8	15	50
	750	92.6	5.0	88.0	0.8	107.9	2.2	104.6	1.8		
	1500	108.3	1.9	92.8	1.7	113.7	3.1	118.8	1.6		
FB_1_	100	107.7	9.0	79.4	7.9	66.1	15.9	93.6	5.8	3	10
	500	94.9	6.4	75.4	3.4	82.7	5.1	98.4	7.6		
	1000	90.5	4.7	85.8	2.4	68.6	3.7	108.4	0.7		
FB_2_	100	70.6	5.4	86.9	3.3	92.8	16.5	65.3	5.4	1.5	5
	500	101.2	8.9	101.1	1.0	84.8	6.9	73.5	5.6		
	1000	85.1	7.4	111.6	9.2	92.5	1.7	80.4	3.0		
T-2	4	85.1	4.3	118.8	2.7	95.3	7.0	101.0	6.9	0.3	1
	20	62.0	3.4	118.7	3.6	77.7	2.6	66.6	12.0		
	40	78.5	14.4	77.5	2.4	89.9	5.1	71.7	2.2		
HT-2	20	89.1	5.2	104.4	5.0	73.8	7.2	82.1	9.6	5	15
	100	84.4	6.3	99.2	12.1	80.4	1.2	84.9	18.3		
	200	75.5	4.2	83.5	11.3	83.5	5.8	93.5	8.4		
OTA	4	96.1	8.4	111.5	3.1	61.5	5.6	71.1	17.3	0.3	1
	20	77.6	4.5	115.8	2.9	73.8	5.0	81.9	3.0		
	40	80.9	4.3	116.5	3.3	87.4	4.4	96.6	3.9		
ZEN	40	80.9	6.2	71.8	2.7	91.8	12.5	74.3	2.3	0.5	1.5
	200	72.3	3.6	78.3	2.5	73.9	2.3	70.7	7.6		
	400	83.9	1.6	77.1	1.6	79.5	9.1	70.7	4.7		
ZAN	40	87.4	2.1	88.0	2.2	82.3	6.6	76.8	11.7	0.5	1.5
	200	92.7	6.4	96.0	1.9	82.0	3.9	76.4	3.3		
	400	72.4	3.6	85.7	1.4	79.1	8.7	71.4	4.2		
α-ZEL	40	78.4	6.5	87.5	5.8	62.1	16.6	87.8	5.0	1	3
	200	92.4	2.7	99.5	7.3	86.6	4.4	79.8	2.2		
	400	88.8	15.3	93.3	2.9	84.6	3.5	82.6	4.5		
β-ZEL	40	83.3	5.8	88.7	9.7	62.3	9.8	65.0	10.2	1	3
	200	79.5	3.9	99.0	10.7	83.8	3.0	84.7	6.9		
	400	94.1	3.6	89.6	2.0	83.6	5.0	84.4	1.3		
β-ZAL	40	66.3	5.4	85.2	7.9	65.8	3.6	63.8	6.0	1	3
	200	110.4	3.4	100.0	9.4	62.3	10.6	99.9	4.9		
	400	93.9	5.1	87.8	2.5	110.8	1.5	87.8	1.7		

**Table 3:** Occurrence and contamination levels of mycotoxins in coix seed, malt, lotus seed, and lilii bulbus.

Mycotoxins	Coix Seed (*n* = 50)				Malt (*n* = 46)			
	>LOD (%)	>MRL ^a^ (%)	Maximum ± SD (μg/kg)	Mean ^b^ (μg/kg)	>LOD (%)	>MRL (%)	Maximum ± SD (μg/kg)	Mean (μg/kg)
AFB_1_	28	6	19.54 ± 2.31	4.55	6.52	6.52	97.00 ± 2.44	36.13
AFB_2_	2	- ^c^	0.55 ± 0.08	0.55	2.17	-	2.04 ± 0.05	2.04
AFG_1_	ND ^d^	-	ND	ND	ND	-	ND	ND
AFG_2_	ND	-	ND	ND	ND	-	ND	ND
DON	18	4	1570.35 ± 4.85	412.36	52.2	2.17	1100.88 ± 11.25	94.06
FB_1_	60	-	1875.77 ± 6.16	193.43	2.17	-	49.65 ± 4.49	49.65
FB_2_	40	-	543.01 ± 5.57	100.61	ND	-	ND	ND
T-2	2	-	15.14 ± 1.13	15.14	6.52	-	56.08 ± 2.68	21.24
HT-2	2	-	32.76 ± 2.45	32.76	2.17	-	260 ± 5.51	260
OTA	12	0	2.10 ± 0.07	1.75	15.22	0	1.88 ± 0.07	1.32
ZEN	100	28	2376.58 ± 9.37	131.85	8.70	4.35	92.02 ± 3.83	46.64
ZAN	12	-	123.17 ± 2.85	40.33	ND	-	ND	ND
α-ZEL	6	-	13.45 ± 0.93	8.56	ND	-	ND	ND
β-ZEL	6	-	12.17 ± 0.59	7.87	ND	-	ND	ND
β-ZAL	ND	-	ND	ND	ND	-	ND	ND
**Mycotoxins**	**Lotus Seed (*n* = 15)**				**Lilii Bulbus (*n* = 16)**			
	**>LOD (%)**	**>MRL (%)**	**Maximum** **(μg/kg)**	**Mean** **(μg/kg)**	**>LOD (%)**	**>MRL (%)**	**Maximum** **(μg/kg)**	**Mean** **(μg/kg)**
AFB_1_	ND	ND	ND	ND	ND	ND	ND	ND
AFB_2_	ND	-	ND	ND	ND	-	ND	ND
AFG_1_	ND	-	ND	ND	ND	-	ND	ND
AFG_2_	13.33	-	1.45 ± 0.03	1.30	ND	-	ND	ND
DON	ND	ND	ND	ND	ND	ND	ND	ND
FB_1_	ND	ND	ND	ND	81.25	-	22.06 ± 2.04	15.29
FB_2_	ND	ND	ND	ND	ND	-	ND	ND
T-2	ND	ND	ND	ND	ND	-	ND	ND
HT-2	ND	ND	ND	ND	37.50	-	267.50 ± 5.81	174.21
OTA	13.33	0	2.03 ± 0.04	1.99	25	6.25	19.3 ± 1.22	7.11
ZEN	ND	-	ND	ND	ND	ND	ND	ND
ZAN	ND	ND	ND	ND	ND	-	ND	ND
α-ZEL	ND	ND	ND	ND	ND	-	ND	ND
β-ZEL	ND	ND	ND	ND	ND	-	ND	ND
β-ZAL	ND	ND	ND	ND	ND	-	ND	ND

^a^ Chinese regulatory limit (national food safety standard/standards for limits of mycotoxins in food/GB2761-2017). ^b^ Mean level of samples > LOD. ^c^ There are no maximum residue limits for this mycotoxin. ^d^ Not detected.

**Table 4:** Exposure of detected mycotoxins in coix seeds, malt, lotus seeds, and lilii bulbus for males and females.

Mycotoxin	PMTDI ^a^ (ng kg^−1^ b.w. day^−1^)	Exposure (ng kg^−1^ b.w. day^−1^)							
		Coix Seed		Malt		Lotus Seed		Lilii Bulbus	
		Male	Female	Male	Female	Male	Female	Male	Female
AFB_1_	− ^b^	2.06	2.38	8.19	9.46	0	0	0	0
AFB_2_	−	0.25	0.29	0.46	0.53	0	0	0	0
AFG_2_	−	0	0	0	0	0.29	0.34	0	0
DON	1000	186.87	215.90	21.31	24.62	0	0	0	0
FB_1_	2000	87.66	101.27	11.25	13.00	0	0	2.77	3.20
FB_2_	2000	45.59	52.67	0	0	0	0	0	0
T-2	60	6.86	7.93	4.81	5.56	0	0	0	0
HT-2	60	14.85	17.15	58.91	68.06	0	0	31.58	36.48
OTA	14	0.79	0.92	0.30	0.35	0.45	0.52	1.29	1.49
ZEN	500	59.75	69.03	10.57	12.21	0	0	0	0
ZAN	500	18.28	21.12	0	0	0	0	0	0
α-ZEL	500	3.88	4.48	0	0	0	0	0	0
β-ZEL	500	3.57	4.12	0	0	0	0	0	0

^a^ Provisional maximum tolerable daily intake (PMTDI) established by the Joint FAO/WHO Expert Committee on Food Additives (JECFA). ^b^ No data.

## Data Availability

The data that support the findings of this study are available from the corresponding author upon reasonable request.

## Supplementary Materials

The following supporting information can be downloaded at: https://www.mdpi.com/article/10.3390/toxins15030209/s1, Figure S1: UHPLC–MS/MS chromatograms of 15 mycotoxins: AFB1 and AFG1, 10 ng/mL; AFB2 and AFG2, 3 ng/mL; DON, 500 ng/mL; FB1, FB2, HT-2, ZEN, ZAN, α- ZEL, β-ZEL and β-ZAL, 200 ng/mL; T-2 and OTA, 20 ng/mL; Table S1: MRM transitions and collision energies for UHPLC–MS/MS analysis of 15 mycotoxins; Table S2: Sampling of coix seeds, malt, lotus seeds, and lilii bulbus from their main producing area in China; Table S3: Concentrations of the mixed standard working solutions of 15 mycotoxins.
