# Supplementary material for: Contamination and Health Risk Assessment of Multiple Mycotoxins in Edible and Medicinal Plants
Source: Toxins (Basel). 2023 Mar 10;15(3):209. doi: 10.3390/toxins15030209 (PMC10056361; doi:10.3390/toxins15030209)
Supplement: Supplementary file 1 [file toxins-15-00209-s001.zip › toxins-2245161-supplementary.pdf]

Table S1. MRM transitions and collision energies for UHPLC–MS/MS analysis of 15 mycotoxins.

Mycotoxin	Precursor ion (m/z)	Product ion (Quantifier, m/z)	CE (V)	Product ion (Qualifier, m/z)	CE (V)	Retention time (min)	ESI mode
AFB_1	313.00	285.00	-24	241.00	-38	4.7	+
AFB_2	315.00	259.00	-30	287.00	-27	4.5	+
AFG_1	329.00	243.00	-29	311.00	-29	4.4	+
AFG_2	331.00	245.00	-31	257.00	-31	4.2	+
DON	297.15	249.10	-11	231.10	-12	2.7	+
FB_1	722.40	352.30	-34	334.15	-41	4.8	+
FB_2	706.40	336.25	-42	318.25	-41	5.3	+
T-2	489.05	245.05	-25	326.95	-22	5.4	+
HT-2	447.10	285.10	-20	105.10	-48	5.1	+
OTA	404.10	239.00	-22	358.10	-13	5.7	+
ZEN	317.10	175.20	24	131.10	29	5.8	−
ZAN	319.37	275.25	22	205.20	23	5.7	−
α-ZEL	319.15	160.15	32	130.05	35	5.7	−
β-ZEL	319.15	160.15	31	130.05	35	5.7	−
β-ZAL	321.15	277.20	23	107.05	34	5.2	−

Table S2. Sampling of Coix Seed, Malt, Lotus Seed and Lilii Bulbus from their main producing area in China.

Province	No. of sample	Mean temperature (°C)	Mean relative humidity (%)	Latitudes (N)
Heilongjiang	9	4.0	70	43°26′~53°33′
Hebei	25	14.2	62	36°01′~42°37′
Gansu	5	14.8	56	32°31′~42°57′
Jiangsu	6	16.8	76	30°45′~35°08′
Anhui	16	17.2	77	29°41′~34°38′
Fujian	5	20.6	77	23°30′~28°22′
Jiangxi	5	17.7	78	24°29′~30°04′
Hunan	10	18.5	79	24°38′~30°08′
Guizhou	32	16.4	80	24°37′~29°13′
Sichuan	5	15.4	79	26°03′~34°19′
Yunnan	9	15.0	74	21°08′~29°15′
Total	127			

Table S3. Concentrations of the mixed standard working solutions of 15 mycotoxins.

**Mycotoxin**	**Concentration (ng/mL)**					
	**1**	**2**	**3**	**4**	**5**	**6**
AFB_1	0.25	0.5	1	2.5	5	10
AFB_2	0.075	0.15	0.3	0.75	1.5	3
AFG_1	0.25	0.5	1	2.5	5	10
AFG_2	0.075	0.15	0.3	0.75	1.5	3
DON	37.5	75	150	375	750	1500
FB_1	5	10	20	50	100	200
FB_2	2.5	5	10	25	50	100
T-2	0.5	1	2	5	10	20
HT-2	2.5	5	10	25	50	100
OTA	0.5	1	2	5	10	20
ZEN	5	10	20	50	100	200
ZAN	5	10	20	50	100	200
α-ZEL	5	10	20	50	100	200
β-ZEL	5	10	20	50	100	200
β-ZAL	5	10	20	50	100	200

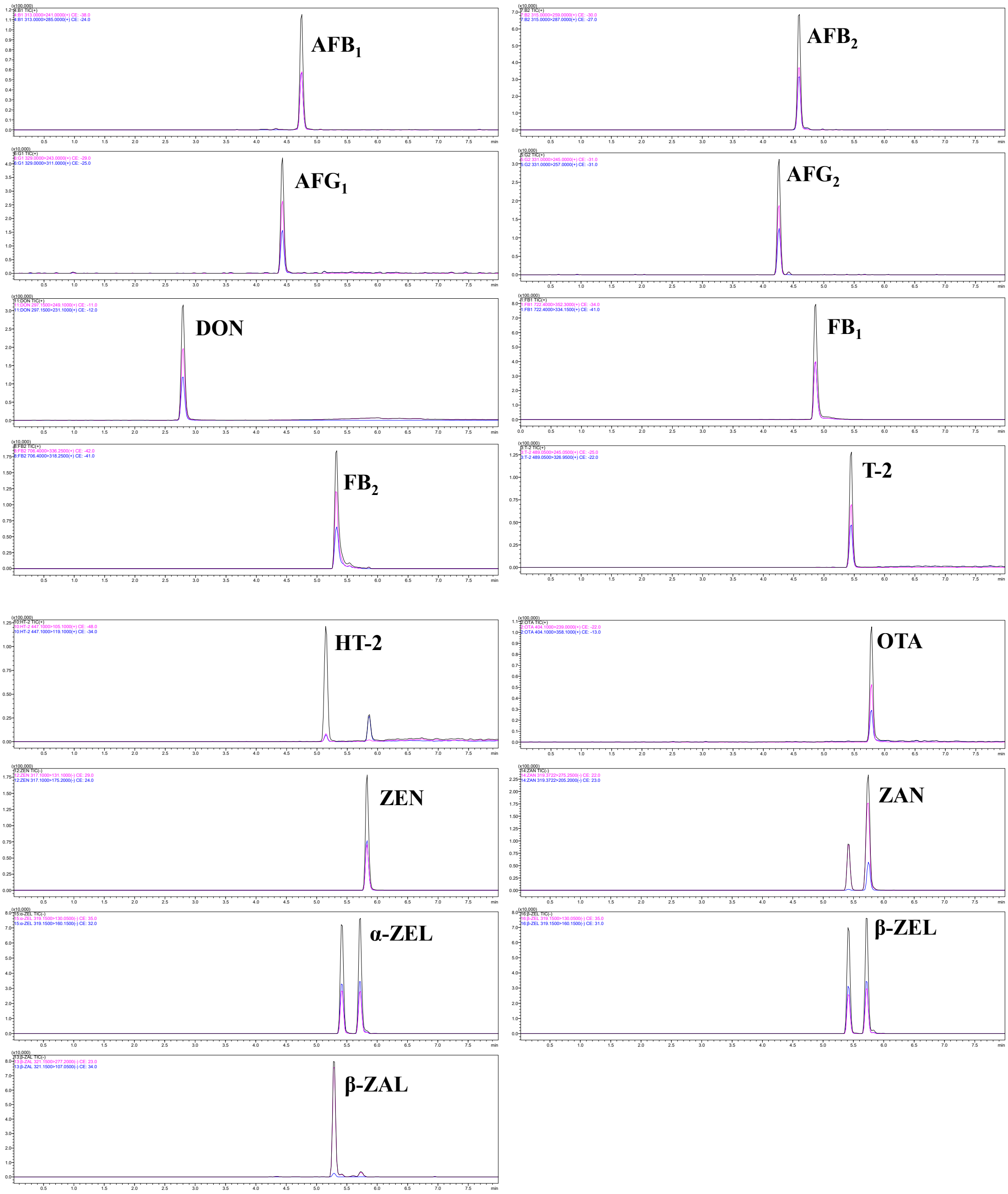

Figure S1. UHPLC–MS/MS chromatograms of 15 mycotoxins: AFB_1 and AFG_1, 10 ng/mL; AFB_2 and AFG_2, 3 ng/mL; DON, 500 ng/mL; FB_1, FB_2, HT-2, ZEN, ZAN, α-ZEL, β-ZEL and β-ZAL, 200 ng/mL; T-2 and OTA, 20 ng/mL.
